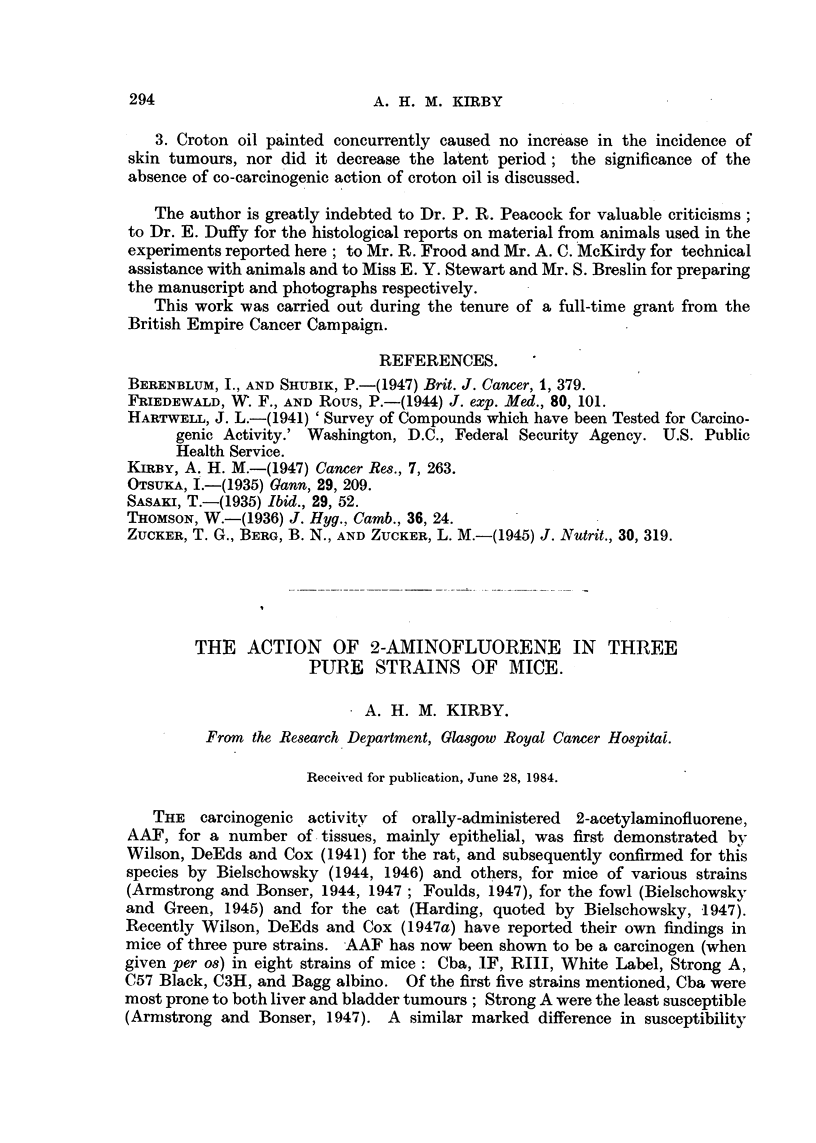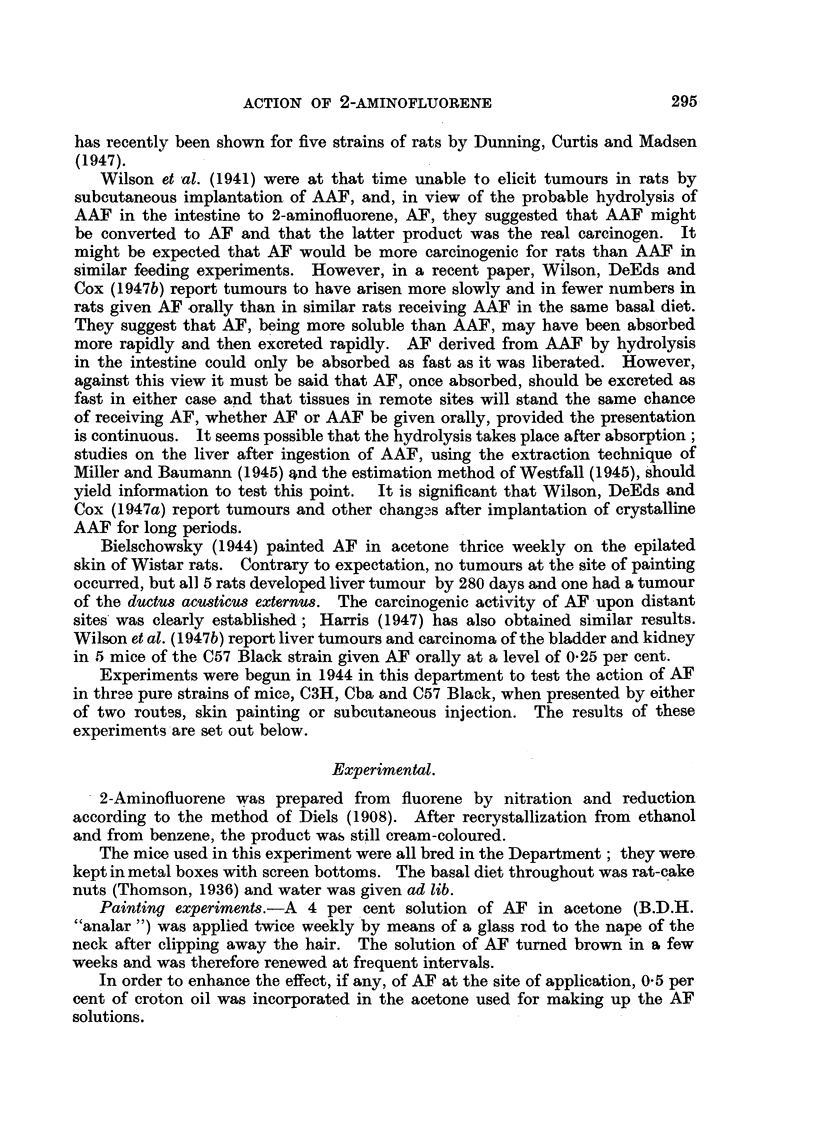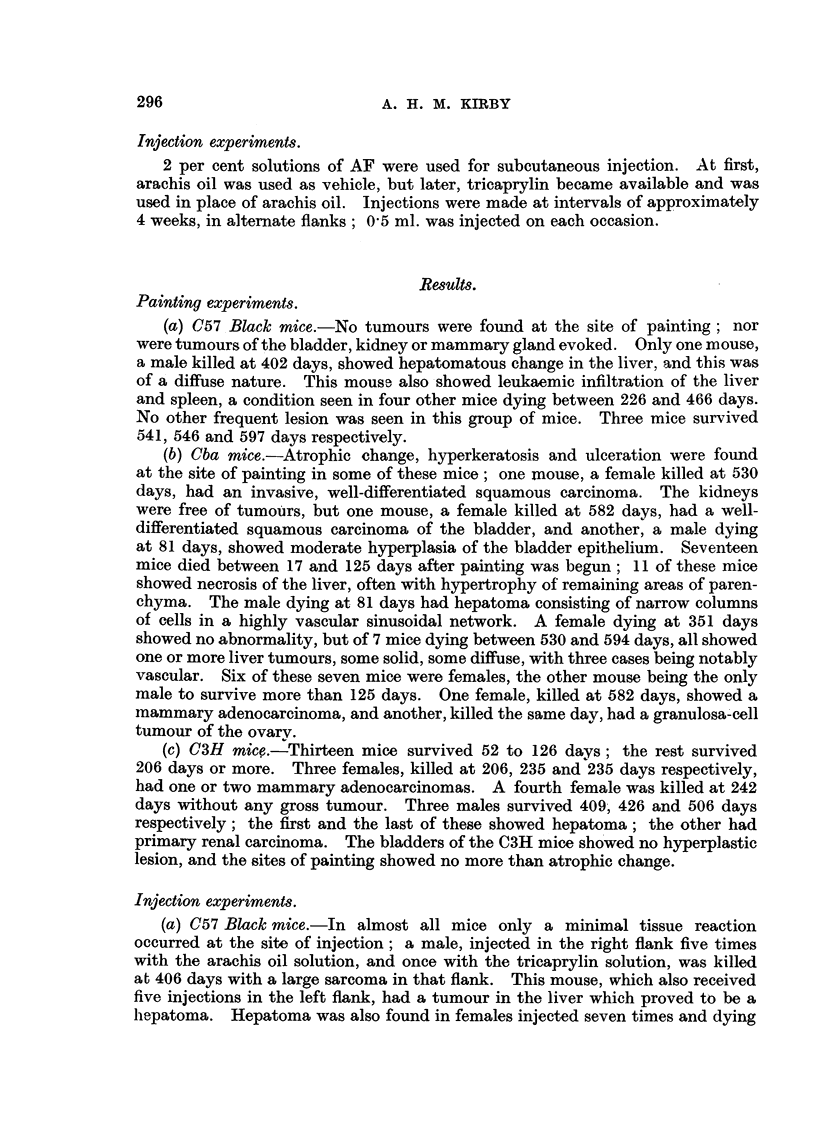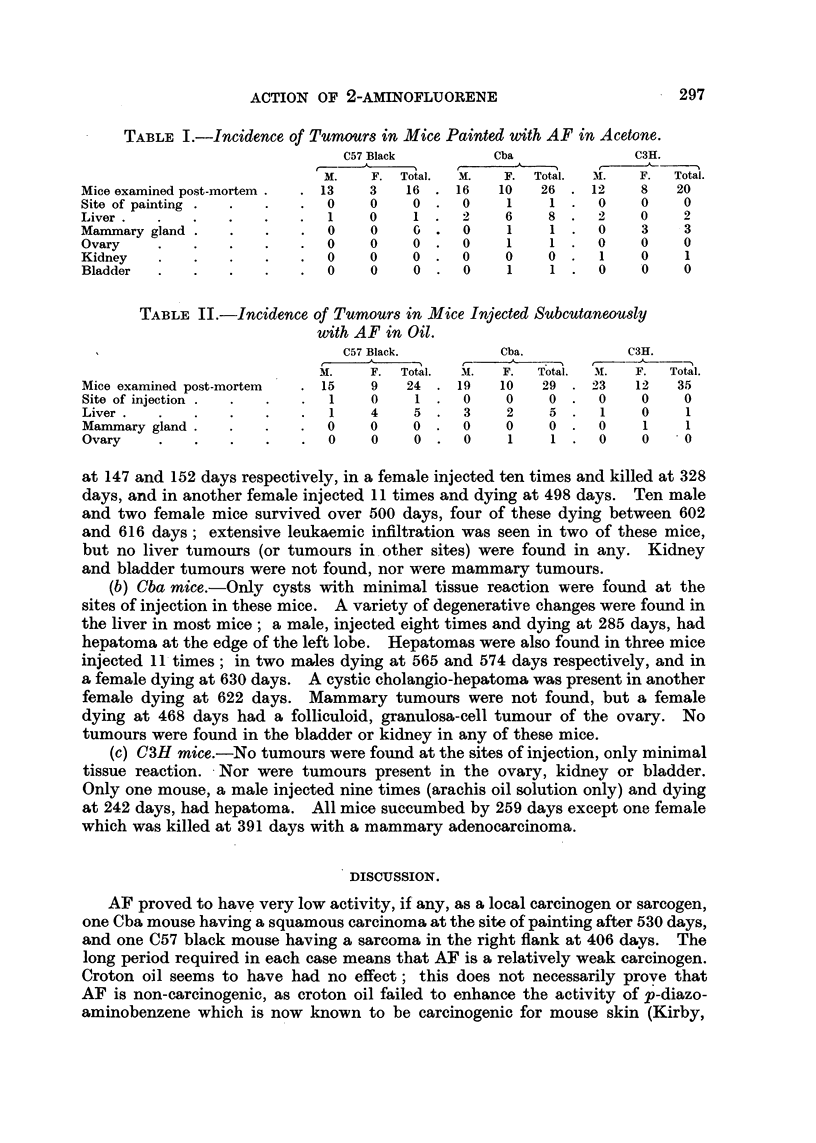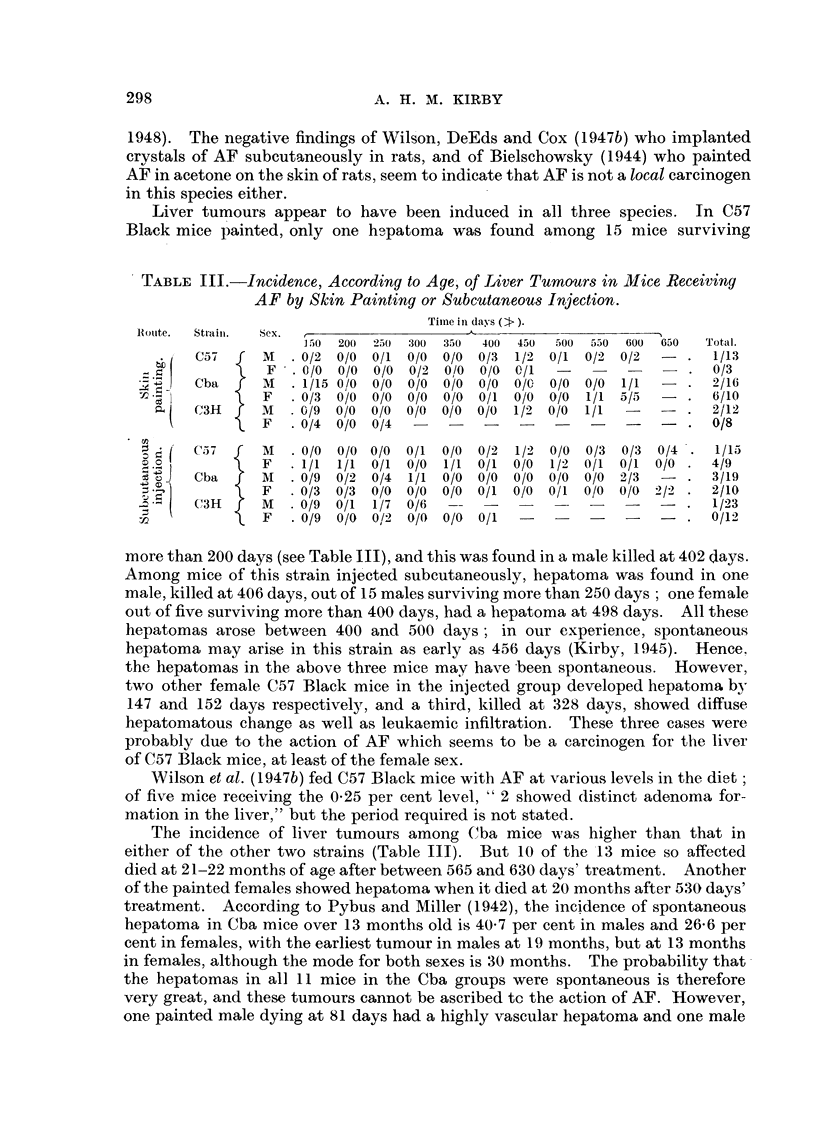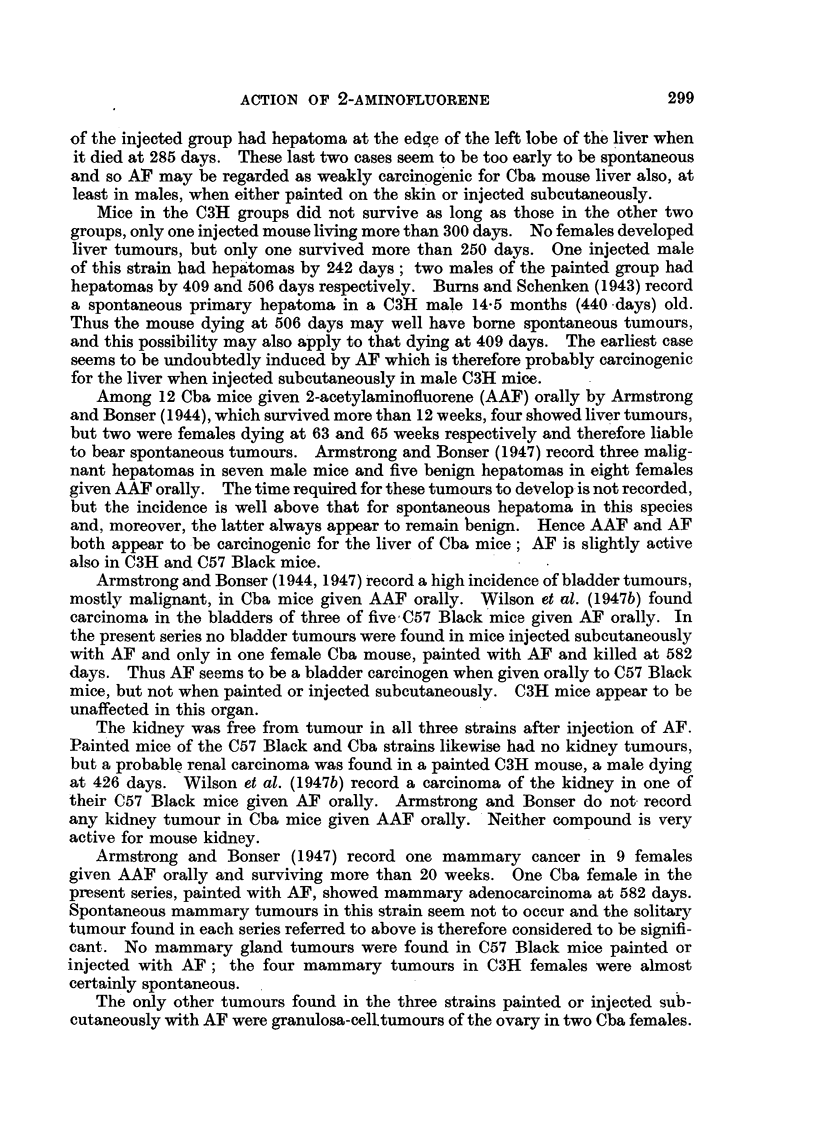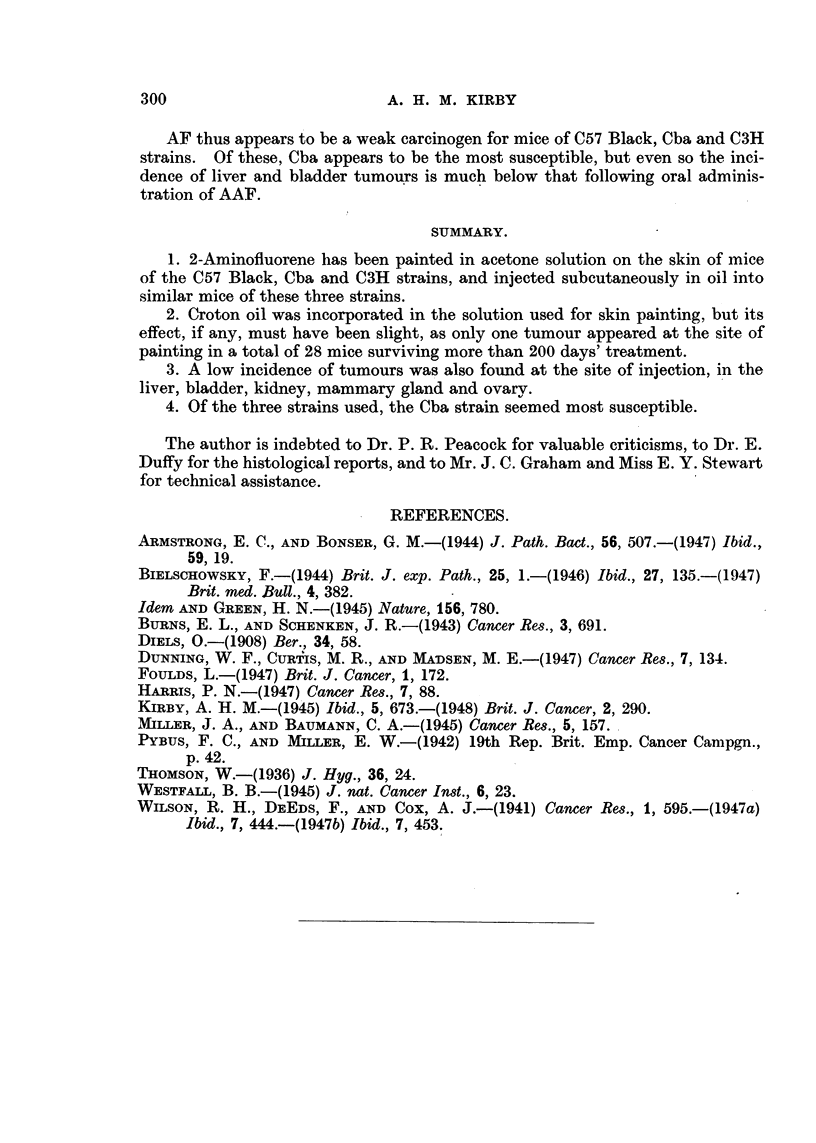# The Action of 2-Aminofluorene in Three Pure Strains of Mice

**DOI:** 10.1038/bjc.1948.35

**Published:** 1948-09

**Authors:** A. H. M. Kirby


### THE ACTION OF 2-AMINOFLUORENE IN THREE PURE STRAINS OF MICE.

A. H. M. KIRBY.

*From the Research Department, Glasgow Royal Cancer Hospital.*

Received for publication, June 28, 1984.

THE carcinogenic activity of orally-administered 2-acetylaminofluorene, AAF, for a number of tissues, mainly epithelial, was first demonstrated by Wilson, DeEds and Cox (1941) for the rat, and subsequently confirmed for this species by Bielschowsky (1944, 1946) and others, for mice of various strains (Armstrong and Bonser, 1944, 1947 ; Foulds, 1947), for the fowl (Bielschowsky and Green, 1945) and for the cat (Harding, quoted by Bielschowsky, 1947). Recently Wilson, DeEds and Cox (1947*a*) have reported their own findings in mice of three pure strains. AAF has now been shown to be a carcinogen (when given *per os*) in eight strains of mice : Cba, IF, RIII, White Label, Strong A, C57 Black, C3H, and Bagg albino. Of the first five strains mentioned, Cba were most prone to both liver and bladder tumours ; Strong A were the least susceptible (Armstrong and Bonser, 1947). A similar marked difference in susceptibility has recently been shown for five strains of rats by Dunning, Curtis and Madsen (1947).

Wilson *et al.* (1941) were at that time unable to elicit tumours in rats by subcutaneous implantation of AAF, and, in view of the probable hydrolysis of AAF in the intestine to 2-aminofluorene, AF, they suggested that AAF might be converted to AF and that the latter product was the real carcinogen. It might be expected that AF would be more carcinogenic for rats than AAF in similar feeding experiments. However, in a recent paper, Wilson, DeEds and Cox (1947*b*) report tumours to have arisen more slowly and in fewer numbers in rats given AF orally than in similar rats receiving AAF in the same basal diet. They suggest that AF, being more soluble than AAF, may have been absorbed more rapidly and then excreted rapidly. AF derived from AAF by hydrolysis in the intestine could only be absorbed as fast as it was liberated. However, against this view it must be said that AF, once absorbed, should be excreted as fast in either case and that tissues in remote sites will stand the same chance of receiving AF, whether AF or AAF be given orally, provided the presentation is continuous. It seems possible that the hydrolysis takes place after absorption; studies on the liver after ingestion of AAF, using the extraction technique of Miller and Baumann (1945) and the estimation method of Westfall (1945), should yield information to test this point. It is significant that Wilson, DeEds and Cox (1947*a*) report tumours and other changes after implantation of crystalline AAF for long periods.

Bielschowsky (1944) painted AF in acetone thrice weekly on the epilated skin of Wistar rats. Contrary to expectation, no tumours at the site of painting occurred, but all 5 rats developed liver tumour by 280 days and one had a tumour of the *ductus acusticus externus*. The carcinogenic activity of AF upon distant sites was clearly established; Harris (1947) has also obtained similar results. Wilson *et al.* (1947*b*) report liver tumours and carcinoma of the bladder and kidney in 5 mice of the C57 Black strain given AF orally at a level of 0·25 per cent.

Experiments were begun in 1944 in this department to test the action of AF in three pure strains of mice, C3H, Cba and C57 Black, when presented by either of two routes, skin painting or subcutaneous injection. The results of these experiments are set out below.

### *Experimental.*

2-Aminofluorene was prepared from fluorene by nitration and reduction according to the method of Diels (1908). After recrystallization from ethanol and from benzene, the product was still cream-coloured.

The mice used in this experiment were all bred in the Department; they were kept in metal boxes with screen bottoms. The basal diet throughout was rat-cake nuts (Thomson, 1936) and water was given *ad lib.*

*Painting experiments.*—A 4 per cent solution of AF in acetone (B.D.H. "analar") was applied twice weekly by means of a glass rod to the nape of the neck after clipping away the hair. The solution of AF turned brown in a few weeks and was therefore renewed at frequent intervals.

In order to enhance the effect, if any, of AF at the site of application, 0·5 per cent of croton oil was incorporated in the acetone used for making up the AF solutions.

*Injection experiments.*

2 per cent solutions of AF were used for subcutaneous injection. At first, arachis oil was used as vehicle, but later, tricaprylin became available and was used in place of arachis oil. Injections were made at intervals of approximately 4 weeks, in alternate flanks ; 0·5 ml. was injected on each occasion.

### *Results.*

*Painting experiments.*

(*a*) *C57 Black mice.*—No tumours were found at the site of painting ; nor were tumours of the bladder, kidney or mammary gland evoked. Only one mouse, a male killed at 402 days, showed hepatomatous change in the liver, and this was of a diffuse nature. This mouse also showed leukaemic infiltration of the liver and spleen, a condition seen in four other mice dying between 226 and 466 days. No other frequent lesion was seen in this group of mice. Three mice survived 541, 546 and 597 days respectively.

(*b*) *Cba mice.*—Atrophic change, hyperkeratosis and ulceration were found at the site of painting in some of these mice ; one mouse, a female killed at 530 days, had an invasive, well-differentiated squamous carcinoma. The kidneys were free of tumours, but one mouse, a female killed at 582 days, had a well-differentiated squamous carcinoma of the bladder, and another, a male dying at 81 days, showed moderate hyperplasia of the bladder epithelium. Seventeen mice died between 17 and 125 days after painting was begun ; 11 of these mice showed necrosis of the liver, often with hypertrophy of remaining areas of parenchyma. The male dying at 81 days had hepatoma consisting of narrow columns of cells in a highly vascular sinusoidal network. A female dying at 351 days showed no abnormality, but of 7 mice dying between 530 and 594 days, all showed one or more liver tumours, some solid, some diffuse, with three cases being notably vascular. Six of these seven mice were females, the other mouse being the only male to survive more than 125 days. One female, killed at 582 days, showed a mammary adenocarcinoma, and another, killed the same day, had a granulosa-cell tumour of the ovary.

(*c*) *C3H mice.*—Thirteen mice survived 52 to 126 days ; the rest survived 206 days or more. Three females, killed at 206, 235 and 235 days respectively, had one or two mammary adenocarcinomas. A fourth female was killed at 242 days without any gross tumour. Three males survived 409, 426 and 506 days respectively ; the first and the last of these showed hepatoma ; the other had primary renal carcinoma. The bladders of the C3H mice showed no hyperplastic lesion, and the sites of painting showed no more than atrophic change.

*Injection experiments.*

(*a*) *C57 Black mice.*—In almost all mice only a minimal tissue reaction occurred at the site of injection ; a male, injected in the right flank five times with the arachis oil solution, and once with the tricaprylin solution, was killed at 406 days with a large sarcoma in that flank. This mouse, which also received five injections in the left flank, had a tumour in the liver which proved to be a hepatoma. Hepatoma was also found in females injected seven times and dying

TABLE I.—*Incidence of Tumours in Mice Painted with AF in Acetone.*

| | C57 Black | | | Cba | | | C3H. | | |
|---|---|---|---|---|---|---|---|---|---|
| | M. | F. | Total. | M. | F. | Total. | M. | F. | Total. |
| Mice examined post-mortem | 13 | 3 | 16 | 16 | 10 | 26 | 12 | 8 | 20 |
| Site of painting | 0 | 0 | 0 | 0 | 1 | 1 | 0 | 0 | 0 |
| Liver | 1 | 0 | 1 | 2 | 6 | 8 | 2 | 0 | 2 |
| Mammary gland | 0 | 0 | 0 | 0 | 1 | 1 | 0 | 3 | 3 |
| Ovary | 0 | 0 | 0 | 0 | 1 | 1 | 0 | 0 | 0 |
| Kidney | 0 | 0 | 0 | 0 | 0 | 0 | 1 | 0 | 1 |
| Bladder | 0 | 0 | 0 | 0 | 1 | 1 | 0 | 0 | 0 |

TABLE II.—*Incidence of Tumours in Mice Injected Subcutaneously with AF in Oil.*

| | C57 Black. | | | Cba. | | | C3H. | | |
|---|---|---|---|---|---|---|---|---|---|
| | M. | F. | Total. | M. | F. | Total. | M. | F. | Total. |
| Mice examined post-mortem | 15 | 9 | 24 | 19 | 10 | 29 | 23 | 12 | 35 |
| Site of injection | 1 | 0 | 1 | 0 | 0 | 0 | 0 | 0 | 0 |
| Liver | 1 | 4 | 5 | 3 | 2 | 5 | 1 | 0 | 1 |
| Mammary gland | 0 | 0 | 0 | 0 | 0 | 0 | 0 | 1 | 1 |
| Ovary | 0 | 0 | 0 | 0 | 1 | 1 | 0 | 0 | 0 |

at 147 and 152 days respectively, in a female injected ten times and killed at 328 days, and in another female injected 11 times and dying at 498 days. Ten male and two female mice survived over 500 days, four of these dying between 602 and 616 days ; extensive leukaemic infiltration was seen in two of these mice, but no liver tumours (or tumours in other sites) were found in any. Kidney and bladder tumours were not found, nor were mammary tumours.

(*b*) *Cba mice.*—Only cysts with minimal tissue reaction were found at the sites of injection in these mice. A variety of degenerative changes were found in the liver in most mice ; a male, injected eight times and dying at 285 days, had hepatoma at the edge of the left lobe. Hepatomas were also found in three mice injected 11 times ; in two males dying at 565 and 574 days respectively, and in a female dying at 630 days. A cystic cholangio-hepatoma was present in another female dying at 622 days. Mammary tumours were not found, but a female dying at 468 days had a folliculoid, granulosa-cell tumour of the ovary. No tumours were found in the bladder or kidney in any of these mice.

(*c*) *C3H mice.*—No tumours were found at the sites of injection, only minimal tissue reaction. Nor were tumours present in the ovary, kidney or bladder. Only one mouse, a male injected nine times (arachis oil solution only) and dying at 242 days, had hepatoma. All mice succumbed by 259 days except one female which was killed at 391 days with a mammary adenocarcinoma.

### DISCUSSION.

AF proved to have very low activity, if any, as a local carcinogen or sarcogen, one Cba mouse having a squamous carcinoma at the site of painting after 530 days, and one C57 black mouse having a sarcoma in the right flank at 406 days. The long period required in each case means that AF is a relatively weak carcinogen. Croton oil seems to have had no effect ; this does not necessarily prove that AF is non-carcinogenic, as croton oil failed to enhance the activity of *p*-diazoaminobenzene which is now known to be carcinogenic for mouse skin (Kirby,

1948). The negative findings of Wilson, DeEds and Cox (1947*b*) who implanted crystals of AF subcutaneously in rats, and of Bielschowsky (1944) who painted AF in acetone on the skin of rats, seem to indicate that AF is not a *local* carcinogen in this species either.

Liver tumours appear to have been induced in all three species. In C57 Black mice painted, only one hepatoma was found among 15 mice surviving

TABLE III.—*Incidence, According to Age, of Liver Tumours in Mice Receiving AF by Skin Painting or Subcutaneous Injection.*

| Route. | Strain. | Sex. | Time in days (≯). 150 | 200 | 250 | 300 | 350 | 400 | 450 | 500 | 550 | 600 | 650 | Total. |
|---|---|---|---|---|---|---|---|---|---|---|---|---|---|---|
| Skin painting. | C57 | M | 0/2 | 0/0 | 0/1 | 0/0 | 0/0 | 0/3 | 1/2 | 0/1 | 0/2 | 0/2 | — | 1/13 |
| | | F | 0/0 | 0/0 | 0/0 | 0/2 | 0/0 | 0/0 | 0/1 | — | — | — | — | 0/3 |
| | Cba | M | 1/15 | 0/0 | 0/0 | 0/0 | 0/0 | 0/0 | 0/0 | 0/0 | 0/0 | 1/1 | — | 2/16 |
| | | F | 0/3 | 0/0 | 0/0 | 0/0 | 0/0 | 0/1 | 0/0 | 0/0 | 1/1 | 5/5 | — | 6/10 |
| | C3H | M | 0/9 | 0/0 | 0/0 | 0/0 | 0/0 | 0/0 | 1/2 | 0/0 | 1/1 | — | — | 2/12 |
| | | F | 0/4 | 0/0 | 0/4 | — | — | — | — | — | — | — | — | 0/8 |
| Subcutaneous injection. | C57 | M | 0/0 | 0/0 | 0/0 | 0/1 | 0/0 | 0/2 | 1/2 | 0/0 | 0/3 | 0/3 | 0/4 | 1/15 |
| | | F | 1/1 | 1/1 | 0/1 | 0/0 | 1/1 | 0/1 | 0/0 | 1/2 | 0/1 | 0/1 | 0/0 | 4/9 |
| | Cba | M | 0/9 | 0/2 | 0/4 | 1/1 | 0/0 | 0/0 | 0/0 | 0/0 | 0/0 | 2/3 | — | 3/19 |
| | | F | 0/3 | 0/3 | 0/0 | 0/0 | 0/0 | 0/1 | 0/0 | 0/1 | 0/0 | 0/0 | 2/2 | 2/10 |
| | C3H | M | 0/9 | 0/1 | 1/7 | 0/6 | — | — | — | — | — | — | — | 1/23 |
| | | F | 0/9 | 0/0 | 0/2 | 0/0 | 0/0 | 0/1 | — | — | — | — | — | 0/12 |

more than 200 days (see Table III), and this was found in a male killed at 402 days. Among mice of this strain injected subcutaneously, hepatoma was found in one male, killed at 406 days, out of 15 males surviving more than 250 days ; one female out of five surviving more than 400 days, had a hepatoma at 498 days. All these hepatomas arose between 400 and 500 days ; in our experience, spontaneous hepatoma may arise in this strain as early as 456 days (Kirby, 1945). Hence, the hepatomas in the above three mice may have been spontaneous. However, two other female C57 Black mice in the injected group developed hepatoma by 147 and 152 days respectively, and a third, killed at 328 days, showed diffuse hepatomatous change as well as leukaemic infiltration. These three cases were probably due to the action of AF which seems to be a carcinogen for the liver of C57 Black mice, at least of the female sex.

Wilson *et al.* (1947*b*) fed C57 Black mice with AF at various levels in the diet ; of five mice receiving the 0·25 per cent level, " 2 showed distinct adenoma formation in the liver," but the period required is not stated.

The incidence of liver tumours among Cba mice was higher than that in either of the other two strains (Table III). But 10 of the 13 mice so affected died at 21–22 months of age after between 565 and 630 days' treatment. Another of the painted females showed hepatoma when it died at 20 months after 530 days' treatment. According to Pybus and Miller (1942), the incidence of spontaneous hepatoma in Cba mice over 13 months old is 40·7 per cent in males and 26·6 per cent in females, with the earliest tumour in males at 19 months, but at 13 months in females, although the mode for both sexes is 30 months. The probability that the hepatomas in all 11 mice in the Cba groups were spontaneous is therefore very great, and these tumours cannot be ascribed to the action of AF. However, one painted male dying at 81 days had a highly vascular hepatoma and one male of the injected group had hepatoma at the edge of the left lobe of the liver when it died at 285 days. These last two cases seem to be too early to be spontaneous and so AF may be regarded as weakly carcinogenic for Cba mouse liver also, at least in males, when either painted on the skin or injected subcutaneously.

Mice in the C3H groups did not survive as long as those in the other two groups, only one injected mouse living more than 300 days. No females developed liver tumours, but only one survived more than 250 days. One injected male of this strain had hepatomas by 242 days ; two males of the painted group had hepatomas by 409 and 506 days respectively. Burns and Schenken (1943) record a spontaneous primary hepatoma in a C3H male 14·5 months (440 days) old. Thus the mouse dying at 506 days may well have borne spontaneous tumours, and this possibility may also apply to that dying at 409 days. The earliest case seems to be undoubtedly induced by AF which is therefore probably carcinogenic for the liver when injected subcutaneously in male C3H mice.

Among 12 Cba mice given 2-acetylaminofluorene (AAF) orally by Armstrong and Bonser (1944), which survived more than 12 weeks, four showed liver tumours, but two were females dying at 63 and 65 weeks respectively and therefore liable to bear spontaneous tumours. Armstrong and Bonser (1947) record three malignant hepatomas in seven male mice and five benign hepatomas in eight females given AAF orally. The time required for these tumours to develop is not recorded, but the incidence is well above that for spontaneous hepatoma in this species and, moreover, the latter always appear to remain benign. Hence AAF and AF both appear to be carcinogenic for the liver of Cba mice ; AF is slightly active also in C3H and C57 Black mice.

Armstrong and Bonser (1944, 1947) record a high incidence of bladder tumours, mostly malignant, in Cba mice given AAF orally. Wilson *et al.* (1947*b*) found carcinoma in the bladders of three of five C57 Black mice given AF orally. In the present series no bladder tumours were found in mice injected subcutaneously with AF and only in one female Cba mouse, painted with AF and killed at 582 days. Thus AF seems to be a bladder carcinogen when given orally to C57 Black mice, but not when painted or injected subcutaneously. C3H mice appear to be unaffected in this organ.

The kidney was free from tumour in all three strains after injection of AF. Painted mice of the C57 Black and Cba strains likewise had no kidney tumours, but a probable renal carcinoma was found in a painted C3H mouse, a male dying at 426 days. Wilson *et al.* (1947*b*) record a carcinoma of the kidney in one of their C57 Black mice given AF orally. Armstrong and Bonser do not record any kidney tumour in Cba mice given AAF orally. Neither compound is very active for mouse kidney.

Armstrong and Bonser (1947) record one mammary cancer in 9 females given AAF orally and surviving more than 20 weeks. One Cba female in the present series, painted with AF, showed mammary adenocarcinoma at 582 days. Spontaneous mammary tumours in this strain seem not to occur and the solitary tumour found in each series referred to above is therefore considered to be significant. No mammary gland tumours were found in C57 Black mice painted or injected with AF ; the four mammary tumours in C3H females were almost certainly spontaneous.

The only other tumours found in the three strains painted or injected subcutaneously with AF were granulosa-cell tumours of the ovary in two Cba females.

AF thus appears to be a weak carcinogen for mice of C57 Black, Cba and C3H strains. Of these, Cba appears to be the most susceptible, but even so the incidence of liver and bladder tumours is much below that following oral administration of AAF.

### SUMMARY.

1. 2-Aminofluorene has been painted in acetone solution on the skin of mice of the C57 Black, Cba and C3H strains, and injected subcutaneously in oil into similar mice of these three strains.

2. Croton oil was incorporated in the solution used for skin painting, but its effect, if any, must have been slight, as only one tumour appeared at the site of painting in a total of 28 mice surviving more than 200 days' treatment.

3. A low incidence of tumours was also found at the site of injection, in the liver, bladder, kidney, mammary gland and ovary.

4. Of the three strains used, the Cba strain seemed most susceptible.

The author is indebted to Dr. P. R. Peacock for valuable criticisms, to Dr. E. Duffy for the histological reports, and to Mr. J. C. Graham and Miss E. Y. Stewart for technical assistance.

### REFERENCES.

ARMSTRONG, E. C., AND BONSER, G. M.—(1944) *J. Path. Bact.*, **56**, 507.—(1947) *Ibid.*, **59**, 19.

BIELSCHOWSKY, F.—(1944) *Brit. J. exp. Path.*, **25**, 1.—(1946) *Ibid.*, **27**, 135.—(1947) *Brit. med. Bull.*, **4**, 382.

*Idem* AND GREEN, H. N.—(1945) *Nature*, **156**, 780.

BURNS, E. L., AND SCHENKEN, J. R.—(1943) *Cancer Res.*, **3**, 691.

DIELS, O.—(1908) *Ber.*, **34**, 58.

DUNNING, W. F., CURTIS, M. R., AND MADSEN, M. E.—(1947) *Cancer Res.*, **7**, 134.

FOULDS, L.—(1947) *Brit. J. Cancer*, **1**, 172.

HARRIS, P. N.—(1947) *Cancer Res.*, **7**, 88.

KIRBY, A. H. M.—(1945) *Ibid.*, **5**, 673.—(1948) *Brit. J. Cancer*, **2**, 290.

MILLER, J. A., AND BAUMANN, C. A.—(1945) *Cancer Res.*, **5**, 157.

PYBUS, F. C., AND MILLER, E. W.—(1942) 19th Rep. Brit. Emp. Cancer Campgn., p. 42.

THOMSON, W.—(1936) *J. Hyg.*, **36**, 24.

WESTFALL, B. B.—(1945) *J. nat. Cancer Inst.*, **6**, 23.

WILSON, R. H., DEEDS, F., AND COX, A. J.—(1941) *Cancer Res.*, **1**, 595.—(1947*a*) *Ibid.*, **7**, 444.—(1947*b*) *Ibid.*, **7**, 453.